# What Shall We Eat?

**Published:** 1879-06

**Authors:** 


### WHAT SHALL WE EAT?

Dr. E. C. Angell, author of a paper in "The Sanitarian" entitled "Alimentation in Health and Disease," would make wheaten food and not beef the basis of alimentation. In a natural and rational system of dietetics wheat and the allied seed foods, including beans, lentils, peas, and rice, must, he holds, take the place now usurped by animal foods, including, besides flesh meats, butter, cheese, eggs, and milk. Next should come the appetizing, juicy fruits, and then plant foods, which are neither seeds nor fruits, and which are generally styled vegetables. After these come the various animal foods, and last of all the stimulating spices, beverages and other food adjuncts. According to Dr. Angell, "the true life giving and mental, moral and physical force producing bread is neither more nor less than sound, ripe wheat when deprived of its thin outer silicious husk, coarsely ground and mixed with water, and subjected to just that degree of kneading and baking which will suffice to prepare it for mastication, insalivation and the subsequent action of the gastric juice." The dough should be kneaded into rolls a little larger than the largest maccaroni, and when baked the product gets the name of "sticks." In these "sticks" we have every nutritious element of the grain, with no fermentation, no cryptogamic vegetation, no deleterious chemical or mineral ingredients. We have, furthermore, a substance that must be chewed, as it can not be swallowed without due mastication and insalivation, and consequently its digestion is insured. Attrition or cold blast wheat, coarsely ground and unbolted, contains all the natural nutritive elements of the wheat. Besides this, it possesses the mechanical properties which distend the intestines, promoting their peristaltic action; it is therefore antidotal to dyspepsia. For children it is specially valuable, and its substitution for common bread, and the use of fruits instead of flesh food, until the deciduous teeth shall have given place to the permanent denture, would be of incalculable benefit and would contribute to the production of good teeth. "The early loss of these organs," says Dr. Angell, "is conclusive evidence that the prevailing system of dietetics is radically wrong."

